# Fused bicyclic piperidines and dihydropyridines by dearomatising cyclisation of the enolates of nicotinyl-substituted esters and ketones

**DOI:** 10.3762/bjoc.6.22

**Published:** 2010-03-02

**Authors:** Heloise Brice, Jonathan Clayden, Stuart D Hamilton

**Affiliations:** 1 School of Chemistry, University of Manchester, Oxford Rd., Manchester M13 9PL, United Kingdom

**Keywords:** bicyclic, cyclisation, dearomatisation, enol ether, heterocycle, pyridine, quinoline

## Abstract

The silyl enol ether derivatives of ketones or esters tethered by a hydrocarbon or ether linkage to the 3-position of a pyridine ring undergo dearomatising nucleophilic attack on the ring once it is activated (as an acylpyridinium species) by the addition of methyl chloroformate. The bicyclic dihydropyridine products are in some cases unstable, but may be isolated after hydrogenation as fused bicyclic piperidines.

## Introduction

Oxidative [1–3] or reductive (nucleophilic) [4–21] dearomatising cyclisation reactions are effective strategies for rapidly building complexity and new reactivity from simple, readily made starting materials. We have used cyclisations of benzamide-stabilised carbanions, for example, to give bicyclic functionalised indolinones as intermediates in the synthesis of the neuroactive amino acids [22–32], while related cyclisations of pyridyl-, nicotinamide- and isonicotinamide-containing carbanions yield related bicyclic dihydropyridines [33–34].

**Scheme 1:**
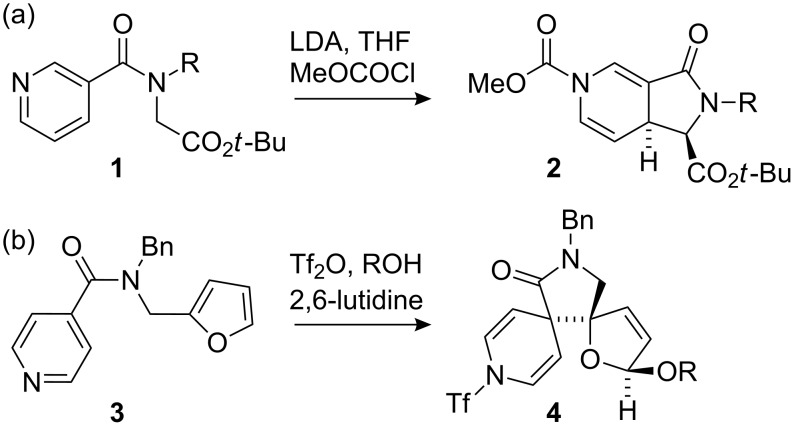
Dearomatising cyclisations (a) of enolates; (b) of electron-rich heteroaromatics.

While reactive carbanions derived from allyl or benzyllithiums will undergo dearomatising addition even into relatively electron rich rings [35–38], the scope of the dearomatisation can be extended to much less reactive nucleophiles with a more electron deficient aromatic acceptor [39–41]. Thus enolates of glycine esters **1** carrying isonicotinoyl or nicotinoyl N-substituents cyclise readily to yield bicyclic amino acid derivatives **2** (Scheme 1a for example) [39]. Even greater reactivity towards intramolecular nucleophilic attack is exhibited by isonicotinamides when activated by N-sulfonation [40–41]. For example, the *N*-furylmethyl isonicotinamide **3** cyclises to the doubly dearomatised bis-spirocycle **4** on treatment with triflic anhydride in the presence of an alcohol [41] (Scheme 1b).

In this paper we report the results of cyclising the enolates of ester and ketones tethered to a nicotinyl nucleus via chains which do not incorporate an amide linkage. The starting materials for these cyclisations do not benefit from the favourable conformational disposition of amides **1** and **3**, making the reactions more challenging. Likewise, the products are evidently less stable than those produced by the reactions in Scheme 1a, but nonetheless they allow new, partially saturated “drug-like” heterocyclic systems to be formed.

## Results and Discussion

### Formation of a carbocyclic ring by dearomatising cyclisation

**Scheme 2:**
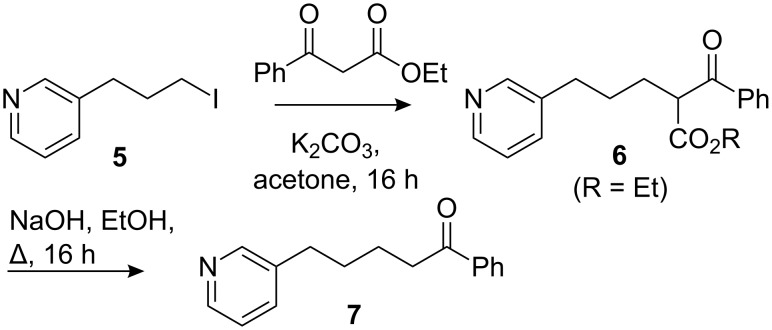
Synthesis of ketone **7**.

The study was initiated with the synthesis of the δ-nicotinyl ketone **7** as illustrated in Scheme 2. Ethyl benzoylacetate was alkylated with 3-(3-iodopropyl)pyridine **5** and the product **6** hydrolysed and decarboxylated to yield the pyridine **7** in moderate yield.

**Scheme 3:**
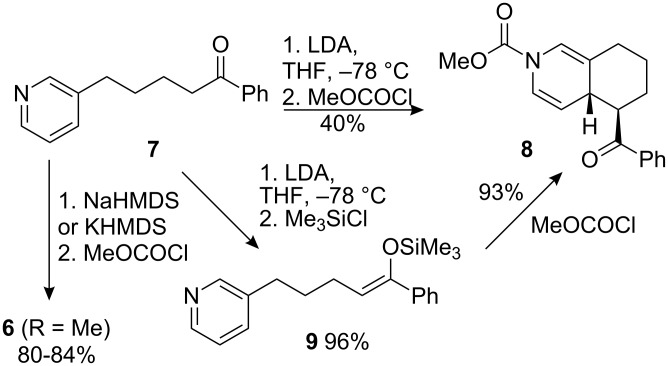
Dearomatising cyclisation to a 5-benzoylhexahydroisoquinoline.

On treatment of **7** with LDA in THF at −78 °C, followed by trapping with methyl chloroformate, a yield of 40% of the bicyclic hexahydroisoquinoline **8** was obtained (Scheme 3), which even after extensive experimentation could not be improved. Lack of crystallinity and overlapping ^1^H NMR signals prevented us from confirming the relative stereochemistry, and the assignment shown in Scheme 3 is on the basis that the benzoyl group of **8** is likely to lie on the *exo* face of the azadecalin system.

We assume, in line with previous results [39], that cyclisation occurs only after the addition of the electrophilic trap (which, precedent suggests, attacks the pyridine lone pair and activates the ring as an acylpyridinium species even in the presence of the lithium enolate). Attempts to use bases with a sodium or potassium counter ion led instead to a high yield of the Claisen product **6** (R = Me), presumably because the sodium and potassium enolates are more reactive than the lithium enolate and compete too well with N-acylation.

We surmised that effective cyclisation onto the acylpyridinium species, avoiding the N- vs. C-acylation problem, might be made possible by decreasing the reactivity of the enolate still further, transforming it into a silyl enol ether **9**. Silyl enol ethers and ketene acetals are known to add effectively to pyridinium species in an intermolecular manner [42–46]. Thus **7** was converted to silyl enol ether **9** in excellent yield under standard conditions. A single geometrical isomer was obtained, presumably *Z* as shown. On treatment with methyl chloroformate, enol ether **9** cyclised to yield **8** again as a single diastereoisomer but in a greatly improved yield of 93%. The strategy of using a less nucleophilic specific enolate equivalent is clearly an effective way of improving selectivity, allowing the chloroformate to activate the pyridine without competing attack by the enolate.

Next we extended the reaction to the cyclisation of a δ-nicotinyl butyrate ester **12** encouraged by the observations of Onaka [47], who demonstrated that silyl ketene acetals can be added (in an intermolecular fashion) to electron deficient pyridines in the presence of trimethylsilyl triflate, tetrabutylammonium fluoride or a montmorillonite clay.

**Scheme 4:**
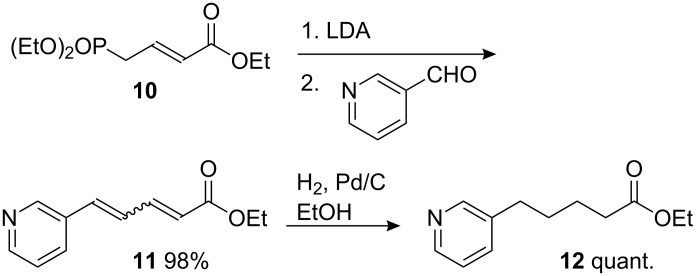
Synthesis of ester **12**.

The cyclisation precursor was synthesised by using the procedure of Hayashi [48] employing a Horner–Wadsworth–Emmons olefination between nicotinaldehyde and phosphonate **10**. The resulting mixture of dienes **11** gave ester **12** after hydrogenation (Scheme 4).

**Scheme 5:**
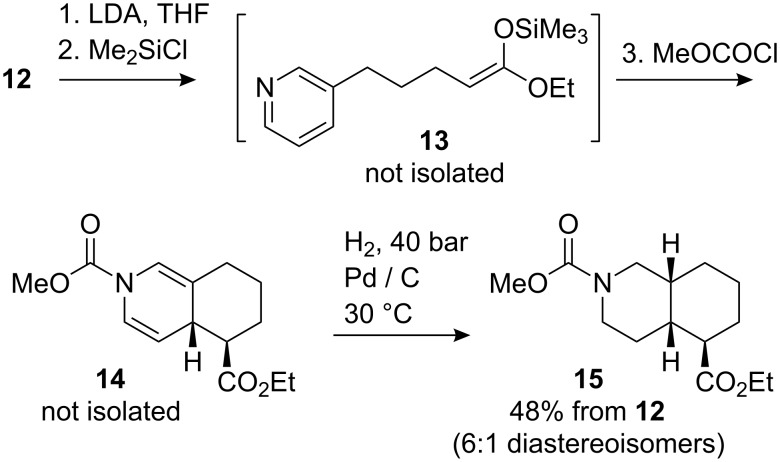
Dearomatising cyclisation of ester **12**.

It proved challenging to isolate cleanly the silyl ketene acetal derived from **12**, so instead we decided to form and cyclise the silyl derivative in a single pot. Thus, ester **12** was added to LDA at −78 °C, and the enolate quenched with trimethylsilyl chloride. After 15 min methyl chloroformate was added and the solution warmed to room temperature. Complete consumption of starting material (by TLC) was accompanied by the appearance of a single less polar spot (*R*_f_ 0.77; EtOAc–petroleum ether 1:1). ^1^H NMR analysis of the crude product after rapid work-up showed two significant sets of new signals at 6.55–6.80 ppm (2H) and 4.65–5.10 ppm (1H) consistent with the dihydropyridine protons of the expected dearomatised product **14** (Scheme 5). However, in contrast with the clean spectra and dearomatised product **8** derived from ketone **7**, duplication of many of the signals in the crude ^1^H NMR spectrum of **14** suggested the existence of either a mixture of diastereoisomers or rotamers caused by restricted rotation of the carbamate group.

No dihydropyridine was isolable from this mixture by flash chromatography, probably due to rapid re-aromatisation. However, immediate hydrogenation at ambient pressure using the conditions developed by Arnott for related 3,4-fused dihydropyridines [39] gave **15** in 45% yield after chromatography as an inseparable mixture of two diastereoisomers in a ratio of approximately 6:1. A slightly improved yield of 48% was obtained by the use of an H-cube flow hydrogenation apparatus at 40 bar and 30 °C.

**Figure 1:**
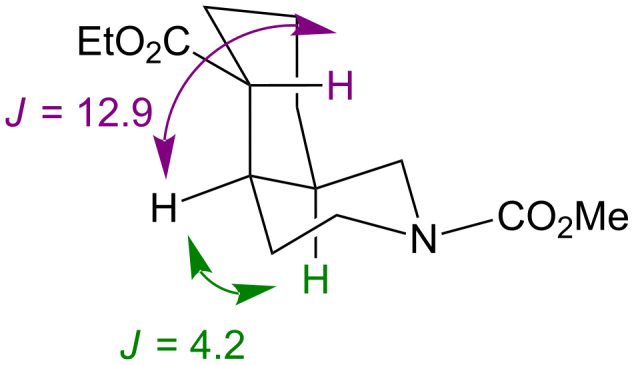
Coupling constants (Hz) in the major diastereoisomer of **15**.

Unfortunately, again the lack of crystallinity and the large number of overlapping signals in the ^1^H NMR spectrum frustrated an unequivocal assignment of the stereochemistry. However, hydrogenation of related fused dihydropyridines has always led to *cis* stereochemistry at the ring junction [32,39]. The consequent expected axial–equatorial relationship between the protons at the ring junction is supported by a coupling constant of 4.2 Hz between these protons in **15** (Figure 1) in the major product diastereoisomer. The corresponding 12.9 Hz coupling to the proton α to the ester group is consistent with adoption of an *exo*–equatorial orientation by this substituent.

### Formation of a tetrahydrofuran by dearomatising cyclisation

**Scheme 6:**
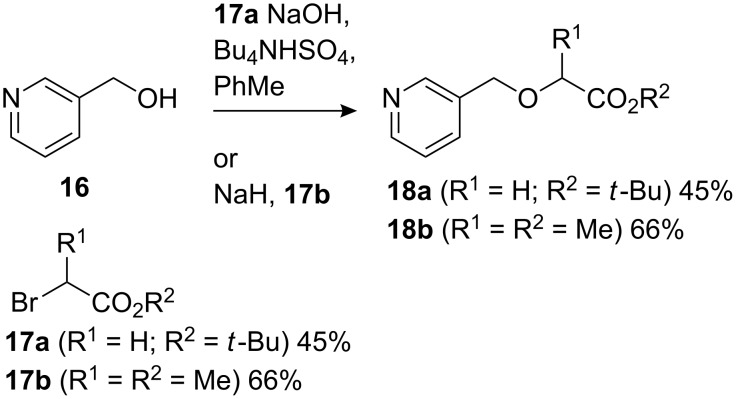
Synthesis of esters **18**.

**Scheme 7:**
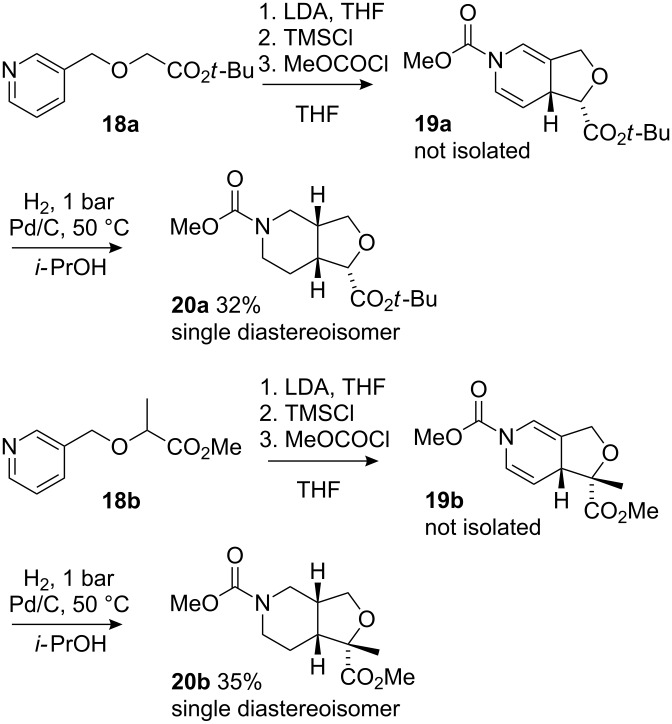
Dearomatising cyclisation to form tetrahydrofurans.

Encouraged by the successful formation of carbocyclic rings in dearomatising cyclisations of nicotinyl ketones and esters, we moved to extend the reaction to the analogous formation of tetrahydofuranyl esters by cyclisation of starting materials incorporating an enolate nucleophile and a nicotinyl electrophile tethered through an ether linkage. Alkylation of 3-hydroxymethylpyridine by *t*-butyl bromoacetate **17a** or bromopropionate **17b** suffered from competing N-alkylation but returned acceptable yields of the esters **18a** and **18b** (Scheme 6). As with **13**, we anticipated that the silyl ketene acetal derivatives **19** would be challenging to isolate, so both starting esters **18a** and **18b** were treated with LDA and Me_3_SiCl followed by methyl chloroformate (Scheme 7). As with **14**, re-aromatisation was fast and the crude products **19** were therefore hydrogenated at atmospheric pressure to give **20a** in up to 32% yield from **18a** and **20b** in up to 35% yield from **18b**. The instability of the two non-isolable intermediates meant however that these yields were not consistently reproducible and yields around 25% were more commonly observed. However, scrupulous avoidance of contact with oxygen before the hydrogenation step improved the yield of **20a** to 41%. Attempted cyclisation without formation of the silyl enol ether (i.e. omitting Me_3_SiCl) led to a complex mixture of products.

**Figure 2:**
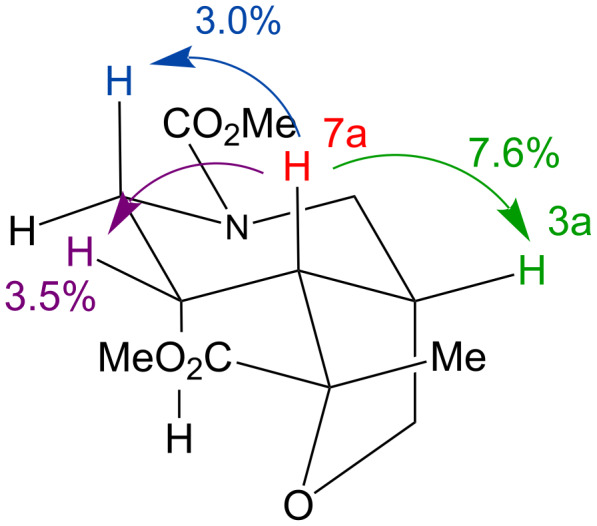
Determination of the stereochemistry of **20b**. Arrows indicate nuclear Overhauser enhancements.

In both cases the cyclic products were obtained as single diastereoisomers, indicating a diastereoselective cyclisation and a face-selective hydrogenation. An nOe experiment on cyclic ether **20b**, irradiating the 7a ring junction proton, showed nOe enhancements of protons 3a, 6 (1H) and 7 (1H) (Figure 2). This result is consistent with a *cis*-fused ring junction. A lack of conclusive nOes prevented determination of the stereochemistry at the ester-bearing centres of **20a** or **20b**. However, a similar cyclisation with an amide tether [39] had resulted in an *endo*-orientated ester substituent, and the stereochemistries of **20** are accordingly shown with the ester orientated *endo*.

## Conclusion

Tethered ketone or ester enolate nucleophiles undergo dearomatising attack on a pyridine ring to yield bicyclic products. Yields are greatest if the enolate is first stabilised as a silyl enol ether, presumably because acylation of the pyridine ring to give the electrophilic acylpyridinium species is cleaner. The bicyclic dihydropyridine products are unstable towards re-aromatisation, but can be isolated in moderate to excellent yield if they are hydrogenated in situ, especially when oxygen is excluded prior to and during the hydrogenation.

## Experimental

### 5-Benzoyl-5,6,7,8-tetrahydro-4a*H*-isoquinoline-2-carboxylic acid methyl ester (**8**)

Methyl chloroformate (0.056 ml, 0.722 mmol) was added to a solution of **9** (45 mg, 0.144 mmol) and triethylamine (0.02 ml, 0.144 mmol) in dichloromethane (5 ml) at −78 °C. After warming to room temperature, dichloromethane was added (30 ml), and the solution washed with water (3 × 10 ml) and brine (10 ml). The combined aqueous layers were extracted with dichloromethane (10 ml) and the combined organic layers dried (Na_2_SO_4_), filtered and concentrated under reduced pressure to furnish a light yellow oil. The oil was purified by flash column chromatography (SiO_2_; petroleum ether–EtOAc 9:1) to yield the title compound as a colourless oil (40 mg, 0.135 mmol, 93%); silica gel TLC *R*_f_ 0.38 (petroleum ether–EtOAc 4:1); IR (thin film) ν_max_ (cm^−1^): 1721 (carbamate C=O), 1677 (ketone C=O); ^1^H NMR (500 MHz, CDCl_3_, δ_H_): 7.80 (2H, d, *J* = 7.5 Hz, Ph-H), 7.41 (1H, t, *J* = 8.0 Hz, Ph-H), 7.32 (2H, t, *J* = 8.0 Hz, Ph-H), 6.67 (1H, br, py-H_2_), 6.54 (1H, br, py-H6), 4.60 (2H, br, py-H_5_) 3.64 (3H, br, OCH_3_), 3.33 (2H, m, py-H_4_ and CH), 2.1–1.3 (6H, br-m, CH_2_); ^13^C NMR (125 MHz, CDCl_3_, δ_C_): 202.9 (ketone C=O), 152.2 (carbamate C=O), 137.3, 133.6, 129.1 and 128.8 (aromatic), 123.4 and 123.0 (py-C_6_), 120.9 and 119.9 (py-C_3_), 117.1 and 116.8 (py-C_2_), 108.1 and 107.7 (py-C_5_), 60.8 (py-C_4_), 53.8 and 53.7 (OCH_3_), 38.0 and 37.8 (CH), 32.5 and 32.4 (CH_2_), 31.7 and 31.6 (CH_2_), 27.5 (CH_2_); CIMS *m/z* (relative intensity): 298 (100%, M+H^+^), 238 (40%, M-CO_2_Me); EIMS *m/z* (relative intensity): 297 (10%, M^+^). [Found: M+H^+^, 298.1436. C_18_H_20_NO_3_ requires 298.1438].

### 5-Ethyl 2-methyl octahydroisoquinoline-2,5-(1*H*) dicarboxylate (**15**)

*n*-Butyllithium (0.36 mL of a 1.8 M solution in hexane) was added to a solution of diisopropylamine (0.11 mL, 0.75 mmol) in THF (15 mL) at 0 °C and the mixture stirred for 15 min before cooling to −78 °C. A solution of ester **12** (0.104 g, 0.5 mmol) in THF (5 mL) and then trimethylsilyl chloride (0.10 mL, 0.75 mmol) were added using a cannula. The solution was stirred at −78 °C for 15 min, methyl chloroformate (0.19 mL, 2.5 mmol) was added and the solution warmed to room temperature. The solution was rapidly added to a saturated sodium hydrogen carbonate solution (30 mL), extracted with EtOAc (2 × 30 mL), dried (MgSO_4_), and concentrated to yield an oil. The crude oil was dissolved in isopropanol (6 mL), and 10% palladium/carbon (0.053 g, 0.05 mmol) was added and the suspension immediately placed under a hydrogen atmosphere. The suspension was warmed to 60 °C for 50 h, filtered through celite and evaporated under reduced pressure. The residue was purified by flash chromatography (SiO_2_; EtOAc–petroleum ether 1:19 to 1:1) to yield the title compound (0.061 g, 45%) as a yellow oil which was approximately a 6:1 mixture of diastereoisomers; *R*_f_ (EtOAc–petroleum ether 1:1) 0.45; IR (film) ν_max_ (cm^−1^): 1730 (C=O ester), 1702 (C=O carbamate); ^1^H NMR (300 MHz, CDCl_3_, δ_H_): 3.95–4.25 (2H, m, 1-H and/or 3-H), 4.13 (2H, q, *J* = 7.5 Hz, C*H*_2_CH_3_), 3.68 (3H, s, OMe_maj_), 3.65 (OMe_min_), 2.85–3.00 (1H, br m, 1-H or 3-H), 2.63–2.76 (1H, br, m, 1-H or 3-H), 2.49 (1H, dt, *J* = 13.0 Hz, 4.0, 5-H), 2.24 (1H, ap dq, *J* = 13.0 Hz, 4.0, CH), 1.83 (1H, dt, *J* = 12.5 Hz, 3.0, CH), 1.65–1.74 (3H, m, CH_2_), 1.57 (1H, dd, *J* = 13.0 Hz, 3.5, CH_2_), 1.37–1.51 (2H, m, CH_2_), 1.17–1.35 (3H, m, CH_2_), 1.25 (3H, t, *J* = 7.5 Hz, CH_2_C*H*_3_); ^13^C NMR (CDCl_3_, δ_C_):174.6 (C=O), 156.7 (C=O), 60.5 (OCH_2_), 52.8 (OMe), 49.9 (CH_2_), 47.0 (COCH), 44.4 (NCH_2_), 40.8 (CH), 37.1 (CH), 37.1 (CH_min_), 30.0 (CH_2_), 25.2 (CH_2_), 24.5 (CH_2min_), 24.0 (CH_2_), 22.1 (CH_2_), 21.5 (CH_2min_), 14.6 (CH_2_*C*H_3_); MS *m/z* (relative intensity): 270 (100%, MH^+^); (Found: MH^+^, 270.1699. C_14_H_24_NO_4_ requires MH^+^, 270.1700).

### 1-*tert*-Butyl 5-methyl hexahydrofuro[3,4-*c*]pyridine-1,5(3*H*)-dicarboxylate (**20a**)

*n*-Butyllithium (0.34 mL of a 1.9 M solution in hexane) was added to a solution of diisopropylamine (0.11 mL, 0.75 mmol) in THF (10 mL) at 0 °C and stirred for 15 min before cooling to −78 °C. A solution of the ester **18a** (0.112 g, 0.5 mmol) in THF (5 mL) was added using a cannula followed by trimethylsilyl chloride (0.10 mL, 0.75 mmol). The solution was then stirred at −78 °C for 45 min, methyl chloroformate (0.19 mL, 2.5 mmol) was added and the solution warmed to room temperature. The solution was rapidly worked-up under a nitrogen atmosphere by addition to saturated sodium hydrogen carbonate solution (30 mL) and extraction with EtOAc (15 mL). 10% Palladium on charcoal (0.053 g, 0.05 mmol) was added and the suspension immediately placed under a hydrogen atmosphere. The suspension was warmed to 45 °C for 18 h, filtered through Celite and evaporated under reduced pressure. The residue was purified by flash chromatography (SiO_2_; EtOAc–petroleum ether 1:4 to 1:1) to yield the title compound (0.058 g, 41%) as white prisms; m.p. 49–51 °C (from Et_2_O); *R*_f_ (EtOAc–petroleum ether 1:1) 0.23; IR (film) ν_max_ (cm^−1^): 1745 (C=O ester), 1705 (C=O carbamate; ^1^H NMR (300 MHz, CDCl_3_, δ_H_): 4.37 (1H, d, *J* = 5.0 Hz, 1-H), 3.95–4.10 (2H, m, 4-H (1H) and 6-H (1H)), 3.98 (1H, t, *J* = 8.5 Hz, OC*H*_a_H_b_), 3.79 (1H, t, *J* = 8.5 Hz, OCH_a_*H*_b_), 3.67 (3H, s, OMe), 3.14–3.28 (1H, m, 4-H), 2.71–2.87 (1H, m, 6-H), 2.44–2.57 (2H, m, 3a-H and 7a-H), 1.50–1.58 (2H, m, 7-H), 1.47 (9H, s, (CH_3_)_3_); ^13^C NMR (CDCl_3_, δ_C_): 169.9 (C=O), 156.2 (C=O), 82.1(*C*(CH_3_)_3_), 81.3 (1-C), 69.2 (3-C), 52.9 (OMe), 42.6 (6-C), 41.7 (4-C), 39.6 (7a-C), 38.3 (3a-C), 28.4 ((CH_3_)_3_), 22.2 (7-C); MS *m/z* (relative intensity): 286 (15%, MH^+^), 230 (100%, MH^+^−C(CH_3_)_3_); (Found: MH^+^, 286.1646. C_14_H_23_NO_5_ requires MH^+^, 286.1649).

### Dimethyl 1-methylhexahydrofuro[3,4-*c*]pyridine-1,5(3*H*)-dicarboxylate (**20b**)

*n*-Butyllithium (0.34 mL of a 1.9 M solution in hexane) was added to a solution of diisopropylamine (0.11 mL, 0.75 mmol) in THF (15 mL) at 0 °C and stirred for 20 min before cooling to −78 °C. A solution of the ester **18b** (0.098 g, 0.5 mmol) in THF (5 mL) was added using a cannula followed by trimethylsilyl chloride (0.10 mL, 0.75 mmol). The solution was then stirred at −78 °C for 15 min and methyl chloroformate (0.19 mL, 2.5 mmol) was added and the solution warmed to room temperature. The solution was rapidly worked-up by addition of saturated sodium hydrogen carbonate solution (30 mL), extracted with EtOAc (2 × 30 mL), dried (MgSO_4_) and evaporated under reduced pressure. The residue was dissolved in propan-2-ol (7 mL), 10% palladium on charcoal (0.053 g, 0.05 mmol) was added and the suspension was immediately placed under a hydrogen atmosphere. The suspension was warmed to 50 °C for 24 h, filtered through celite, washed with EtOAc (5 × 10 mL) and evaporated under reduced pressure. The residue was purified by flash chromatography (SiO_2_; EtOAc–petroleum ether 3:17 to 1:1) to yield the title compound (0.033 g, 35%) as a colourless oil; *R*_f_ (EtOAc–petroleum ether 1:1) 0.25; IR (film) ν_max_ (cm^−1^): 1752 (C=O ester), 1702 (C=O carbamate); ^1^H NMR (300 MHz, CDCl_3_, δ_H_): 4.07 (1H, t, *J* = 8.5 Hz, OC*H*_a_H_b_), 3.81 (1H, t, *J* = 9.0 Hz, OCH_a_*H*_b_), 3.75–4.02 (2H, m, 4-H and 6-H), 3.75 (3H, s, OMe), 3.24 (1H, br d, *J* = 12.5 Hz, 4-H or 6-H), 2.79 (1H, t, *J* = 12.0 Hz, 4-H or 6-H), 2.66 (1H, br s, 3a-H), 2.17–2.27 (1H, m, 7a-H), 1.49–1.64 (2H, m, 7-H), 1.46 (3H, s, (CH_3_); ^13^C NMR (CDCl_3_, δ_C_): 174.0 (C=O ester), 156.4 (C=O carbamate), 87.5 (1-C), 69.1 (3-C), 53.0 (OMe), 52.4 (OMe), 45.1 (7a-C), 42.6 (6-C), 41.8 (4-C), 36.9 (3a-C), 25.1 (C*C*H_3_), 23.6 (7-C); MS *m/z* (relative intensity): 258 (100%, MH^+^), 275 (55%, MNH_4_^+^); (Found: MH^+^, 258.1340. C_12_H_19_NO_5_ requires MH^+^, 258.1336).

## Supporting Information

File 1 Synthesis and characterisation data of starting materials
