# Supplementary material for: Fused bicyclic piperidines and dihydropyridines by dearomatising cyclisation of the enolates of nicotinyl-substituted esters and ketones
Source: Beilstein J Org Chem. 2010 Mar 2;6:22. doi: 10.3762/bjoc.6.22 (PMC2874404; doi:10.3762/bjoc.6.22)
Supplement: File 1 — Synthesis and characterisation data of starting materials [file Beilstein_J_Org_Chem-06-22-s001.pdf]

Supporting Information

for

Fused bicyclic piperidines and dihydropyridines by dearomatising cyclisation of the enolates of nicotinyl-substituted esters and ketones

Heloise Brice, Jonathan Clayden* and Stuart D. Hamilton

School of Chemistry, University of Manchester, Oxford Rd., Manchester M13 9PL, United Kingdom

Email: Jonathan Clayden - clayden@man.ac.uk

Synthesis and characterisation data of starting materials

1-Phenyl-5-(pyridin-3-yl)pentan-1-one (**7**)

Methanesulfonyl chloride (22 mmol, 1.70mL) was added to a stirred solution of 3-pyridine propanol (20 mmol, 2.74 g) and triethylamine (22 mmol, 3.09mL) in dichloromethane (100mL) at 0 °C. After 30 min, the reaction mixture was diluted with dichloromethane (100 mL), washed with water (3 × 60 mL) and brine (60 mL), then dried ($MgSO_4$), filtered, and carefully concentrated under reduced pressure (solidification indicates polymerisation) to yield a colourless oil. The resulting oil was dissolved in acetone (100 mL) and sodium iodide was added (22 mmol, 3.30 g). This solution was stirred under reflux for 2 h. The white precipitate was removed by filtration, the solvent removed under reduced pressure, and dichloromethane added (200 mL). The solution was washed with 10% sodium thiosulfate solution (60 mL), water (2 × 60 mL) and brine (60 mL), then dried ($MgSO_4$), filtered and carefully concentrated under reduced pressure to yield **5** as a brown oil (solidification indicates polymerisation). This oil was dissolved in acetone (100 mL). 3-Oxo-3-phenylpropanoate (22 mmol, 3.81 mL) was added followed by potassium carbonate (24 mmol, 3.32 g) and the mixture heated under reflux overnight. The solvent was removed under reduced pressure and the product **6** was decarboxylated by heating under reflux in a solution of 2 M sodium hydroxide (60 mL) and ethanol (30 mL) for 4 h. The ethanol was removed under reduced pressure, dichloromethane was added (200 mL), and the solution was washed with water (3 × 80 mL) and brine (80 mL). The combined aqueous layers were re-extracted with dichloromethane (80 mL) and the combined organic layers dried ($MgSO_4$), filtered and concentrated under reduced pressure to give a brown oil. Purification by flash column chromatography (SiO_2, 1:1 petrol:EtOAc) gave the title compound as a pale yellow crystalline solid (1.62 g, 6.78 mmol, 34%); m.p. 44–47 °C; silica gel TLC R_f 0.17 (1:1 petrol:EtOAc); ν_{max}/cm^{-1} (thin film) 1685 (C=O); δ_H (300 MHz; $CDCl_3$) 8.49 (2H, br, py-H_2 and py-H_6), 8.01–7.95 (2H, m, Ph-H), 7.62–7.45 (4H, m, Ph-H and py-H_4), 7.24 (1H, dd, *J* 8 and 5, py-H_5), 3.03 (2H, d, *J* 7, CH_2), 2.70 (2H, d, *J* 7, CH_2),

1.89–1.69 (4H, m, CH_2); δ_C (75 MHz; $CDCl_3$) 200.2 (C=O), 150.1, 147.5, 137.7, 137.2. 136.2, 133.3, 128.9, 128.3 and 123.6 (aromatic), 38.5, 33.2, 30.1 and 24.0 (CH_2); *m/z* (CI) 240 (100%, M+H$^+$); *m/z* (EI) 239 (5%, M$^+$), 105 (45%, PhCO), 77 (100%, C_6H_5). [Found: M$^+$, 239.1307. $C_{16}H_{17}NO$ requires 239.1305].

3-((Z)-5-Phenyl-5-trimethylsilanyloxy-pent-4-enyl)-pyridine (**9**)

n-Butyllithium solution (1.1 mmol) was added to a stirred solution of diisopropylamine (1.1 mmol, 0.16 mL) in THF (15 mL) at 0 °C. After 10 min, the solution was cooled to –78 °C and a solution of the ketone **7** (1.0 mmol, 239 mg) in THF (5 mL) was added via cannula. After 10 min, chlorotrimethylsilane (1.2 mmol, 0.15 mL) was added and the solution allowed to warm to room temperature. Diethyl ether was added (60 mL) and the organic phase washed with sat. sodium hydrogen carbonate solution (20 mL), water (2 × 20 mL) and brine (20 mL). The combined aqueous layers were extracted with diethyl ether (20 mL), the combined organic layers dried (Na_2SO_4), filtered and concentrated under reduced pressure to give a light yellow oil. Purification by flash column chromatography (SiO_2, 4:1 petrol:EtOAc) afforded the title compound as a colourless oil (299 mg, 0.96 mmol, 96%); silica gel TLC R_f 0.51 (1:1 petrol:EtOAc); ν_{max}/cm^{-1} (thin film) 1648 (C=C), 1252 ($SiMe_3$), 1057 (Si–O), 844 ($SiMe_3$); δ_H (300 MHz; $CDCl_3$) 8.52 (1H, s, py-H_2), 8.48 (1H, d, *J* 5, py-H_6), 7.56 (1H, d, *J* 8, py-H_4), 7.51–7.47 (2H, m, Ph-H), 7.38–7.22 (4H, m, Ph-H and py-H_3), 5.28 (1H, t, *J* 7, CH), 2.72 (2H, t, *J* 8, CH_2), 2.28 (2H, dt, *J* 8 and 8, CH_2), 1.80 (2H, tt, *J* 8 and 8, CH_2), 0.14 (9H, s, $Si(CH_3)_3$); δ_C (75 MHz; $CDCl_3$) 150.3, 149.9, 147.5, 139.3, 137.9, 136.2, 128.3, 127.8, 125.6, 123.5 and 110.6 (aromatic and C=C), 33.0, 31.4 and 25.9 (CH_2), 0.8 ($Si(CH_3)_3$); *m/z* (CI) 312 (80%, M+H$^+$), 240 (100%, hydrolysed). [Found: M$^+$, 311.1257. $C_{19}H_{25}NOSi$ requires 311.1705].

2-Benzoyl-5-pyridin-3-yl-pentanoic acid methyl ester (**6**) (R = Me)

Potassium hexamethyldisilazide (0.275 mmol) was added to a solution of 1-phenyl-5-(pyridin-3-yl)pentan-1-one (60 mg, 0.25 mmol) in THF (10 mL) at –78 °C. After stirring for 1 h at –78 °C, methyl chloroformate was added (0.021 mL, 0.275 mmol) and the solution allowed to warm to room temperature. Ether was added (30 mL), and the solution washed with water (3 × 10 mL) and then brine (10 mL). The combined aqueous layers were then re-extracted with ether (10 mL) and the combined organic layers dried (Na_2SO_4), filtered, and concentrated under reduced pressure to give a brown oil. Purification by flash column chromatography (SiO_2; 7:3 petrol:EtOAc) gave the title compound as a colourless oil (62 mg, 0.209 mmol, 84%); silica gel TLC R_f 0.30 (1:1 petrol:EtOAc); ν_{max}/cm^{-1} (thin film) 1762 (C=O); δ_H (500 MHz; $CDCl_3$) 8.45 (2H, br, py-H_2 and py-H_6), 7.54-7.23 (7H, Ph-H, py-H_3 and py-H_5), 5.71 (1H, t, *J* 7, CH), 3.77 (3H, s, OCH_3), 2.64 (2H, t, *J* 8, CH_2), 2.19 (2H, q, *J* 7, CH_2), 1.72 (2H, m, CH_2); δ_C (75 MHz; $CDCl_3$) 153.9, 149.1, 147.5, 146.6, 137.3, 134.8, 128.9, 128.7, 124.7, 124.0 and 117.6 (C=O and aromatic), 55.8 (OCH_3), 32.8, 30.4 and 25.6 (CH_2); *m/z* (CI, NH_4^+) 298 (100%, M+H$^+$). [Found: M+H$^+$, 297.1360. $C_{18}H_{19}NO_3$ requires 297.1359].

Ethyl 5-(Pyridin-3'-yl)penta-2,4-dienoate (**11**) [1]

n-Butyllithium (2.89 mL of a 1.9 M solution in hexane) was added to a solution of diisopropylamine (0.81 mL, 5.75 mmol) in THF (17 mL) at −78 °C and the mixture stirred for 20 min before addition of triethyl phosphonocrotonate (1.27 mL, 5.75 mmol). After a further 15 min 3-pyridinecarboxaldehyde (0.48 mL, 5 mmol) was added and the solution warmed to 0 °C over 90 min. Acetic acid (0.5 mL) was added and the solution concentrated under reduced pressure. The resulting oil was treated with saturated sodium hydrogen carbonate solution (30 mL), and the solution extracted with EtOAc (3 x 20 mL). The combined extracts were dried ($MgSO_4$) and concentrated under reduced pressure.

The residue was purified by flash chromatography (SiO_2; EtOAc-petrol 3:7 to 1:1) to yield the title compound (1.00 g, 98%) as a colourless oil; R_f (EtOAc-petrol 1:1) 0.41; ν_{max}(film)/cm^{-1} 1708 (C=O); δ_H (300 MHz; $CDCl_3$) 8.67 (1H, s, py2'-H), 8.52 (1H, d, *J* 5.0, py6'-H), 7.78 (1H, dt, *J* 8.0, 2.0, py4'-H), 7.43 (1H, dd, *J* 15.0, 9.0, pyCHC*H*), 7.29 (1H, dd, *J* 8.0, 5.0, py5'-H), 6.82-6.99 (2H, m, pyCH and COCHC*H*), 6.03 (1H, d, *J* 15.5, COCH), 4.23 (2H, q, *J* 7.0, OCH_2), 1.23 (3H, t, *J* 7.0, CH_3); δ_c ($CDCl_3$) 167.0 (C=O), 149.9 (py6'-C), 149.1 (py2'-C), 143.9 (pyCH*C*H), 136.4 (COCH*C*H), 133.6 (py4'-C), 132.1 (py3'-C), 128.5 (py5'-C), 123.0 (CO*C*H), 60.8 (CH_2), 14.6 (CH_3); m/z 204 (100%, MH^+).

Ethyl 5-(Pyridin-3'-yl)pentanoate (**12**) [1].

10% Palladium on charcoal (0.139 g, 0.013 mmol) was added to a solution of diene **11** (1.00 g, 4.82 mmol) in ethanol (24 mL) and the mixture stirred under a hydrogen atmosphere (1 bar) for 1 hour, filtered through celite and washed with ethanol. The filtrate was evaporated under reduced pressure to yield the title compound (1.051 g, 99%) as a colourless oil which was used without further purification. R_f (EtOAc:petrol 1:1) 0.44; ν_{max}(film)/cm^{-1} 1733 (C=O); δ_H (300 MHz; $CDCl_3$) 8.43 (2H, m, py2'-H and py6'-H), 7.48 (1H, dt, *J* 8.0, 2.0, py4'-H), 7.19 (1H, dd, *J* 8.0, 5.0, py5'-H), 4.11 (2H, q, *J* 7.0, OCH_2), 2.62 (2H, t, *J* 8.0, $pyCH_2$), 2.33 (2H, t, *J* 8.0, $COCH_2$), 1.67 (2H, m, CH_2), 1.64 (2H, m, CH_2), 1.23 (3H, t, *J* 7.0, CH_3); δ_C($CDCl_3$) 173.7 (C=O), 150.1 (py2'-C), 147.6 (py6'-C), 137.6 (py3'-C), 136.1 (py4'-C), 123.6 (py5'-C), 60.6 (OCH_2), 34.3 (CO*C*H_2), 32.9 ($pyCH_2$), 30.8 ($pyCH_2$*C*H_2), 24.7 ($COCH_2$*C*H_2), 14.5 (CH_3); m/z 208 (100%, MH^+), (Found: MH^+, 208.1327. $C_{12}H_{17}NO_2$ requires MH^+, 208.1332).

tert-Butyl 2-((pyridin-3'-yl)methoxy)acetate (**18a**) [2]

tert-Butyl bromoacetate (**17a**) (0.29 mL, 1.38 mmol), tetrabutylammonium bisulfate (0.016 g, 0.46 mmol) and aqueous sodium hydroxide (50% w/v, 1 mL) were added to a colourless

solution of 3-pyridinylcarbinol **16** (0.100 g, 0.917 mmol) in toluene (5 mL) and stirred vigorously for 16 h. The solution was diluted with brine (5 mL) and ether (5 mL), and aqueous hydrochloric acid (6N) added until the pH was neutral. The solution was extracted with diethyl ether (3 x 5 mL) and evaporated under reduced pressure. The resulting orange oil was purified by flash chromatography (SiO_2; EtOAc-petrol 1:3 to 1:1) to yield the title compound (0.092 g, 45%) as an orange oil. R_f (EtOAc:petrol 1:1) 0.29; ν_{max}(film)/cm^{-1} 2978 (CH), 1743 (C=O); δ_H (300 MHz; $CDCl_3$) 8.59 (1H, s, 2'-H), 8.55 (1H, d, *J* 5.0, 6'-H), 7.76 (1H, dt, *J* 8.0, 2.0, 4'-H), 7.30 (1H, dd, *J* 8.0, 5.0, 5'-H), 4.63 (2H, s, pyCH_2), 4.01 (2H, s, CH_2CO), 1.48 (9H, s, $(CH_3)_3$); δ_C ($CDCl_3$) 169.5 (C=O), 149.4 (2'-C), 149.2 (6'-C), 136.2 (4'-C), 133.3 (3'-C), 123.8 (5'-C), 82.2 (*C*$(CH_3)_3$), 70.9 (CH_2), 68.3 (CH_2), 28.4 ($(CH_3)_3$); m/z 224 (100%, MH^+).^{1}H-NMR data consistent with literature (^{13}C-NMR data has not been reported).

Methyl 2-((Pyridin-3'-yl)methoxy)propanoate (**18b**)

Sodium hydride (60% dispersion in oil, 0.220 g, 5.50 mmol) was added in two portions to a stirred solution of 3-pyridylcarbinol **16** (0.44 mL, 4.58 mmol) in THF (5 mL) at 0 °C. After 10 min methyl-2-bromopropionate (**17b**) (0.51 mL, 4.58 mmol) was added and solution warmed to room temperature, stirred for 72 h and poured into saturated sodium hydrogen carbonate solution (10 mL). Water (5 mL) was added and the solution extracted with EtOAc (3 x 10 mL). The combined extracts were dried ($MgSO_4$) and evaporated under reduced pressure. The residue was purified by flash chromatography (SiO_2; EtOAc-petrol 1:4 to 1:1) to yield the title compound (0.540 g, 66%) as a colourless oil. R_f (EtOAc:petrol 1:1) 0.21; ν_{max}(film)/cm^{-1} 1748 (C=O); δ_H (300 MHz; $CDCl_3$) 8.58 (1H, d, *J* 2.0, py2'-H), 8.54 (1H, dd, *J* 5.0, 1.5, py6'-H), 7.74 (1H, dt, *J* 8.0, 2.0, py4'-H), 7.29 (1H, dd, *J* 8.0, 5.0, py5'-H), 4.69 (1H, d, *J* 12.0, pyCH_aH_b), 4.47 (1H, d, *J* 12.0, pyCH_aH_b), 4.09 (1H, q, *J* 7.0, CH), 3.76 (3H, s, OMe), 1.45 (3H, ap d, *J* 7.0, CCH_3); δ_C ($CDCl_3$) 173.6 (C=O), 149.5 (py2'-C or

py6'-C), 149.4 (py2'-C or py6'-C), 136.0 (py4'-C), 133.3 (py3'-C), 123.7 (py5'-C), 74.7 ($CHCH_3$), 69.7 (CH_2), 52.3 (OMe), 18.9 (CH_3); m/z 196 (100%, MH^+), (Found: MH^+, 196.0971. $C_{10}H_{12}NO_3$ requires MH^+, 196.0968).

1. Tanouchi, T.; Kawamura, M.; Ohyama, I.; Kajiwara, I.; Iguchi, Y.; Okada, T.; Miyamoto, T.; Taniguchi, K.; Hayashi, M. *J. Med. Chem.* **1981**, *24*, 1149.

2. Kempf, B.; Hampel, N; Ofial, A. R.; Mayr, H. *Chem. Eur. J.* **2003**, *9*, 2209
